# Expanding instrumented gait testing in the community setting: A portable, depth-sensing camera captures joint motion in older adults

**DOI:** 10.1371/journal.pone.0215995

**Published:** 2019-05-15

**Authors:** Robert J. Dawe, Lei Yu, Sue E. Leurgans, Timothy Truty, Thomas Curran, Jeffrey M. Hausdorff, Markus A. Wimmer, Joel A. Block, David A. Bennett, Aron S. Buchman

**Affiliations:** 1 Rush Alzheimer’s Disease Center, Rush University Medical Center, Chicago, Illinois, United States of America; 2 Department of Diagnostic Radiology and Nuclear Medicine, Rush University Medical Center, Chicago, Illinois, United States of America; 3 Department of Neurological Sciences, Rush University Medical Center, Chicago, Illinois, United States of America; 4 Department of Orthopedic Surgery, Rush University Medical Center, Chicago, Illinois, United States of America; 5 Center for Study of Movement, Cognition and Mobility, Neurological Institute, Tel Aviv Sourasky Medical Center, Tel Aviv, Israel; 6 Department of Physical Therapy, Sackler Faculty of Medicine, and Sagol School of Neuroscience, Tel Aviv University, Tel Aviv, Israel; 7 Department of Internal Medicine, Division of Rheumatology, Rush University Medical Center, Chicago, Illinois, United States of America; Geffen School of Medicine at UCLA, UNITED STATES

## Abstract

**Background:**

Currently, it is not feasible to obtain laboratory-based measures of joint motion in large numbers of older adults. We assessed the utility of a portable depth-sensing camera for quantifying hip and knee joint motion of older adults during mobility testing in the community.

**Methods:**

Participants were 52 older adults enrolled in the Rush Memory and Aging Project, a community-based cohort study of aging. In a subset, we compared dynamic hip and knee flexion/extension obtained via the depth-sensing camera with that obtained concurrently using a laboratory-based optoelectronic motion capture system. Then we recorded participants’ annual instrumented gait assessment in the community setting with the depth-sensing camera and examined the inter-relationships of hip and knee range of motion (ROM) with mobility metrics derived from a wearable sensor and other mobility-related health measures.

**Results:**

In the community, we successfully acquired joint motion from 49/52 participants using the depth-sensing camera. Hip and knee ROMs were related to diverse sensor-derived metrics of mobility performance (hip: Pearson’s r = 0.31 to 0.58; knee: Pearson’s r = 0.29 to 0.51), as well as daily physical activity, conventional motor measures, self-report hip and knee pain and dysfunction, mobility disability, and falls.

**Conclusions:**

The depth-sensing camera’s high rate of successful data acquisition and correlations of its hip and knee ROMs with other mobility measures suggest that this device can provide a cost-efficient means of quantifying joint motion in large numbers of community-dwelling older adults who span the health spectrum.

## Introduction

Numerous studies have demonstrated the utility of motion-tracking technologies for elucidating the biomechanics and gait patterns associated with various types of mobility impairments across much of the lifespan [1,2]. However, these technologies are typically available only in specially equipped laboratories at major medical centers or research institutions, not in community hospitals or clinics. Many older adults, particularly those who are physically frail or have cognitive impairments, are unwilling or unable to undergo such assessments due to the required travel [3], as well as the testing burden itself. Repeated testing to obtain longitudinal data is also impractical. These testing limitations have led to critical gaps in our knowledge regarding gait biomechanics across the full health spectrum of older adults and their relation to mobility impairments.

Recent advances in technology now offer new, innovative means of quantifying multiple dimensions of mobility in the community setting. For example, portable gait mats [4] and wearable devices incorporating electronic accelerometers and gyroscopes [5] now allow objective assessment of spatiotemporal features of gait and other mobility performances in large numbers of community-dwelling older adults. Typically, however, joint motion of older adults is not examined as part of gait assessment in the community setting. Newly available depth-sensing cameras, shown in laboratory studies to be accurate in capturing certain aspects of gait [6–9] are portable, inexpensive, and user-friendly and can therefore potentially be used to record three-dimensional body motion of older adults during instrumented mobility testing in the community, from which joint motion metrics can be extracted.

The purpose of this study was to assess the utility of a portable, depth-sensing camera system for three-dimensional motion tracking of older adults in the community setting to obtain clinically relevant metrics of lower extremity joint motion during gait. We first verified that the depth-sensing camera captured joint motion similar to that obtained in a state-of-the-art motion analysis laboratory. We then deployed the depth-sensing camera in the community setting, where we recorded three-dimensional gait motion data from older adults during a uniform, structured mobility testing session. After extracting metrics of hip and knee joint motion, we assessed their potential clinical relevance by examining their correlations with quantitative mobility metrics derived from recordings of a wearable sensor during the same testing session, and also with a diverse range of other mobility-related physical function and health measures.

## Materials and methods

### Participants

Community-dwelling, ambulatory older adults without dementia were enrolled from the Rush Memory and Aging Project (MAP), a longitudinal clinical-pathologic study of chronic conditions of aging that recruits from retirement communities in northeastern Illinois [10]. To participate in MAP, individuals must be free of known dementia at the time of enrollment, agree to annual structured examinations, and sign an Anatomical Gift Act for autopsy at the time of death. MAP participants provided written consent at enrollment, and as part of this pilot study, we obtained additional written consent for video-based motion capture and questionnaires concerning hip and knee pain and dysfunction. The Institutional Review Board of Rush University Medical Center approved this study. In addition, the individual in the figures gave written informed consent (as outlined in the PLOS consent form) to publish the series of video frames.

### Assessment of joint motion with a depth-sensing camera

#### Hardware, data collection and processing, and testing

**Fig 1:**
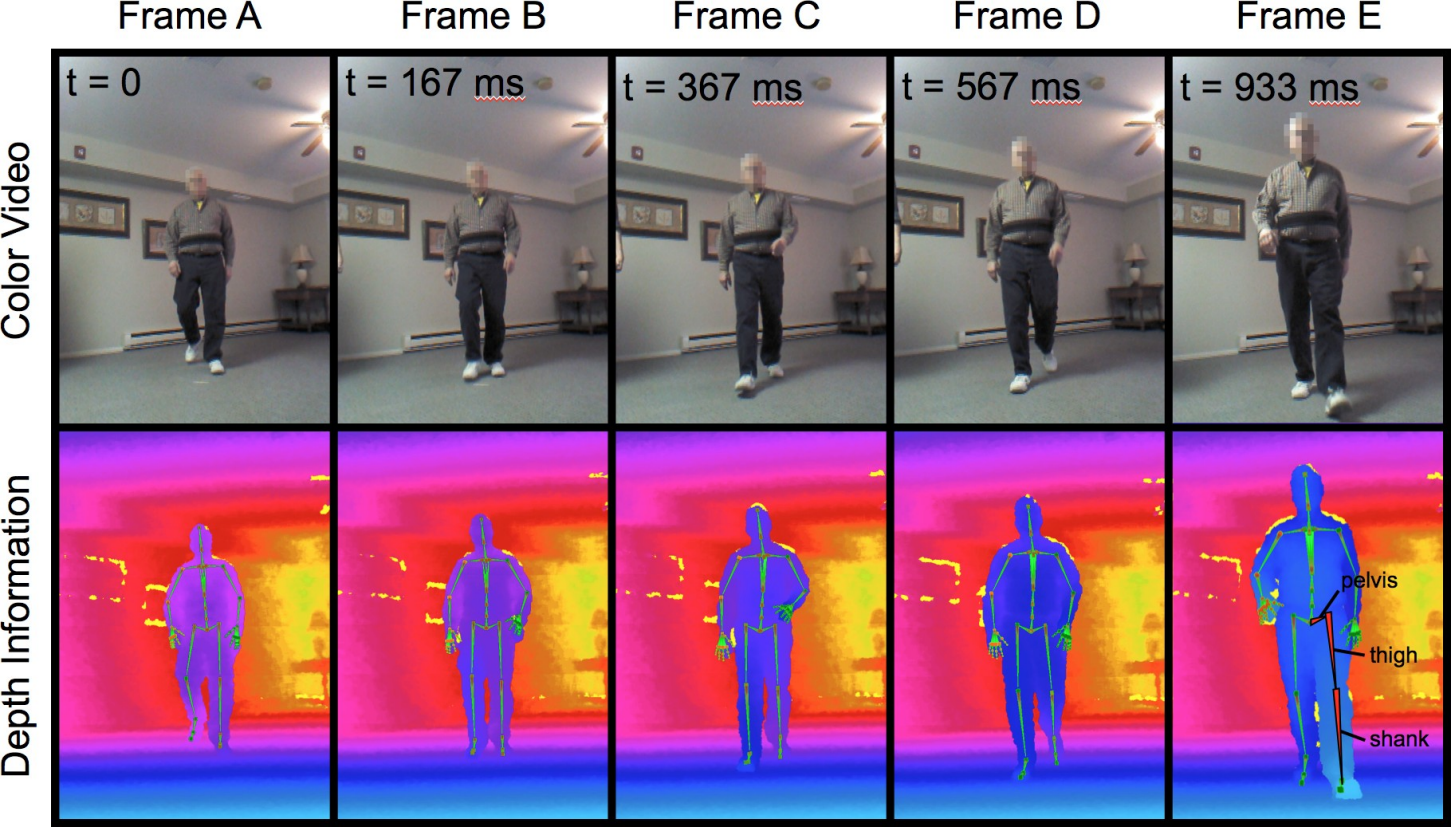
Frames from color video and depth data collected using a depth-sensing camera during structured mobility testing in the community setting. Times (t) are computed based on frame number and indicate the time elapsed since Frame A, the right leg toe-off. In the depth frames, colors code for distance of the object in that pixel from the camera. The green stick figure superimposed on the depth data represents the skeleton of the body pose estimated using motion capture software. The red-highlighted segments in Frame E represent the pelvis, thigh, and shank segments, from which we extracted hip and knee angles. The individual in this figure gave written informed consent (as outlined in the PLOS consent form) to publish this series of video frames.

**Fig 2:**
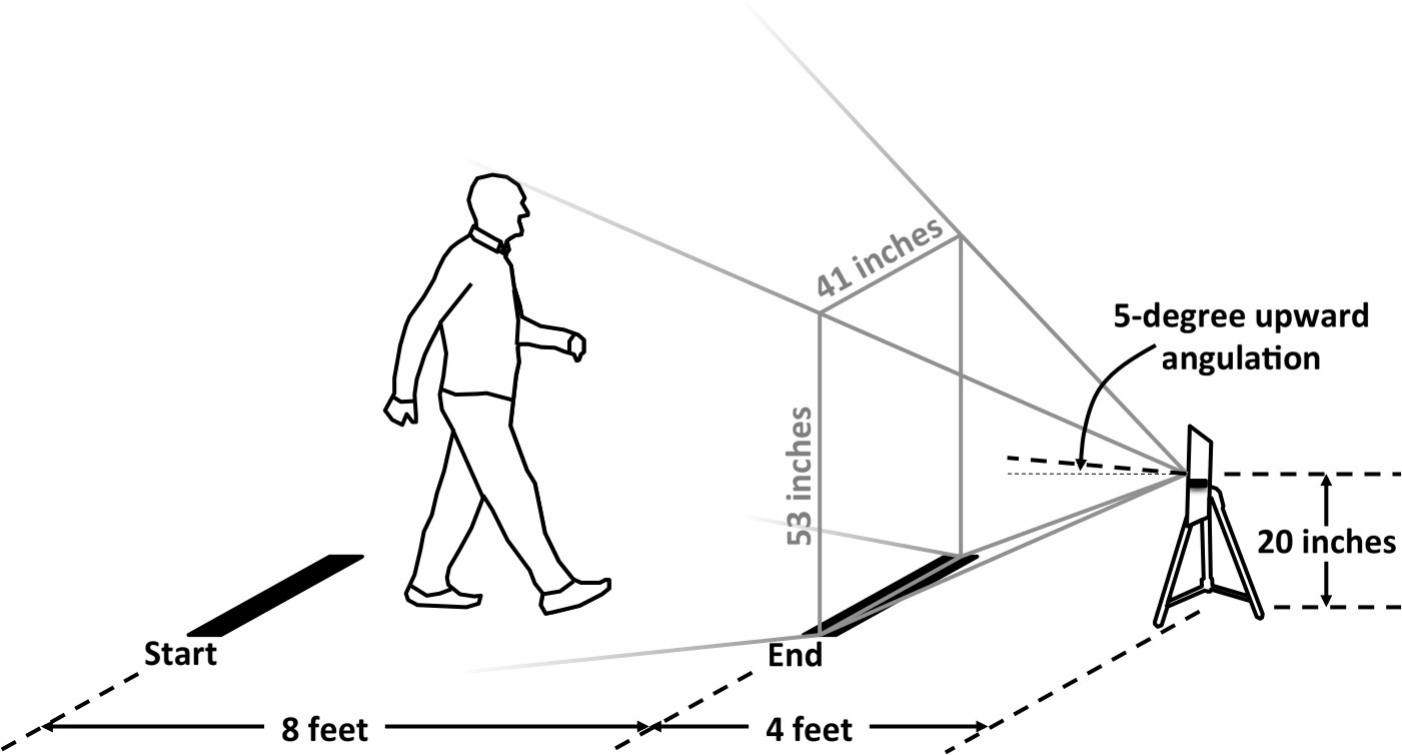
Positioning of the depth-sensing camera with respect to the designated walking course. Participants were asked to walk between two points marked by masking tape on the ground 8 feet apart. The depth-sensing camera was positioned in line with these points, 4 feet beyond one end of the walking course. The camera’s field of view is depicted by gray lines.

A portable, depth-sensing video camera originally developed for video gaming (Kinect for Windows, Microsoft Corporation, Redmond, WA) was employed to capture participants’ biomechanics during gait. Using infrared light, this device records “depth” video, which quantifies an object’s distance from the camera at 30 frames per second (Fig 1). The camera was positioned as shown in Fig 2. Its recording was controlled via software (iPi Recorder version 3.1.1.34, iPi Soft, Moscow, Russia) running on a notebook computer (Pavilion 13 x360 Convertible PC, HP, Palo Alto, CA) as participants traversed a walking path (Fig 1). We transferred this data to a workstation (Z620, HP) to run frame-by-frame body pose estimation software (iPi Mocap Studio version 3.1.2.177, iPi Soft) (Fig 1). In order to compare hip and knee motion captured using the depth-sensing camera with that obtained using a state-of-the-art optoelectronic motion capture system (Qualysis, Gothenborg, Sweden), we first enrolled 10 participants who were able to travel to and undergo testing in the Motion Analysis Laboratory at Rush University Medical Center. These two systems reconstructed similar patterns of hip and knee flexion and extension throughout the gait cycle (S1 File); more of the variation in the tracked joint angles was attributable to between-person sources than stemmed from trial-to-trial differences (within person) or differences between the two systems (depth-sensing camera vs. lab-based system) (S1 File). Additional details of motion data collection, processing, and testing are presented in S1 File.

### Motion capture in the community setting

We then proceeded with testing in the community setting. At their places of residence, participants underwent the annual MAP structured mobility testing protocol, which included a 32-foot walking performance consisting of four traversals of an 8-foot path separated by 180-degree turns, as described in earlier work [11]. Using the techniques outlined above, we recorded the 32-foot walk with the depth-sensing camera. For the two traversals during which the participant was moving toward the camera, we extracted the left and right hip and knee ranges of motion (ROM) computed as the difference between their maximum flexion and extension angles within a gait cycle, as further detailed in S1 File We averaged the four measurements (left and right legs from each of two traversals), yielding a mean hip ROM and a mean knee ROM for each participant.

### Assessment of mobility with a wearable sensor

We also instrumented the structured mobility testing protocol in MAP with a sensor affixed to the lower back. This sensor records acceleration along and rotation rate around each of three orthogonal axes, from which we extracted objective metrics of mobility from the 32-foot walk, the Timed Up and Go (TUG), and the 20-second standing with eyes closed performances. We combined correlated metrics into composite summary scores capturing participants’ gait and balance abilities, as in previous work [11,12].

### Other mobility-related physical function and health measures

We collected other mobility-related health measures during the same annual MAP testing cycle, as previously described. Age at the time of mobility testing was computed based on the participant’s self-reported date of birth recorded at MAP study entry along with sex and years of education. Objective measures of physical activity were derived from a wrist-worn activity monitor [13]. A summary motor measure was computed based on 10 conventional tests of gait, dexterity, and strength [14]. We assessed parkinsonian gait, a relatively common clinical phenotype that is likely to affect objective measures of lower limb kinematics, using a modified version of the United Parkinson’s Disease Rating Scale [15]. BMI was calculated from height and weight [16]. Hip and knee joint pain and dysfunction were assessed via the Hip disability and Osteoarthritis Outcome Survey (HOOS) and the Knee injury and Osteoarthritis Outcome Survey (KOOS) [17,18]. Other data gleaned via participants’ self-report included mobility disability based on the Rosow-Breslau scale [19,20], fall history [21], vascular disease and exposure to vascular disease risk factors [22,23], and frequency or duration of participants’ engagement in specific physical, social, and cognitively stimulating activities [24–26]. Expanded descriptions of the methods by which these data were collected and computed are included in S1 File.

### Statistical analysis

We examined the correlations between hip and knee ROMs and age, sex, education, and the sensor-derived mobility metrics obtained during the same mobility testing session. In further analyses we examined the relationship of joint motion metrics with other mobility-related physical function and health measures that were obtained during the same annual testing cycle and are described in S1 File. We computed the Pearson correlation coefficient between each pair of measures examined and reported the two measures as being correlated if their correlation coefficient met the threshold for statistical significance, set at p<0.05. Statistical analyses were programmed in SAS 9.3 for Linux.

## Results

### Descriptive data

All 52 individuals who we approached consented to the study, and all but 2 completed gait testing while recorded by the depth-sensing camera in the community setting. Of those 50, we failed to extract reliable hip and knee ROMs for one participant due to their unusual gait pattern in which one leg obscured the other from view of the camera during key portions of the gait cycle. The remaining 49-person sample included individuals with a range of self-selected gait speeds (mean = 84 cm/s, SD = 25, min = 35, max = 122). Their joint motion measures, mobility metrics, and other characteristics are summarized in Table 1.

**Table 1:** Joint motion and other characteristics of older adults in this study.

Variable	Mean (SD) or N (%)
**Demographics**	
Age at gait testing (years)	81.4 (8.4)
Female	40 (81.6%)
Education (years)	16.3 (2.8)
**Hip and knee motion (depth-sensing camera)**	
Hip range of motion (degrees)	36.2 (7.4)
Knee range of motion (degrees)	49.4 (6.4)
**Mobility measures (wearable sensor on lower back)**	
Gait speed (composite of z-scores)	0.24 (0.66)
Gait cadence (composite of z-scores)	0.23 (1.05)
Gait step variability (composite of z-scores)	0.03 (1.13)
Gait stride regularity (composite of z-scores)	0.22 (0.85)
Transition 1 duration (composite of z-scores)	0.66 (0.76)
Transition 1 range (composite of z-scores)	0.50 (0.81)
Transition 1 median (composite of z-scores)	0.13 (0.87)
Transition 2 jerk (composite of z-scores)	0.20 (0.78)
Transition 2 range (composite of z-scores)	0.33 (0.71)
Turn yaw (composite of z-scores)	0.15 (0.92)
Turn frequency (composite of z-scores)	-0.02 (0.88)
Standing with eyes closed sway (composite of z-scores)	-0.31 (0.81)
**Actigraphy (wrist-worn monitor)**	
Total daily physical activity (counts/day÷10^5^)	1.69 (0.82)
**Other conventional motor and mobility-related clinical measures**	
Composite motor measure (composite of z-scores)	1.00 (0.23)
Parkinsonian gait (square root of score out of 100; higher = more signs)	8.2 (10.9)
Body mass index (kg/m^2^)	27.0 (4.6)
Mobility disability (Rosow-Breslau scale 0 to 3; higher = more disability)	0.44 (0.74)
Fall history (one or more in the past year)	21 (42.9%)
**Hip disability and Osteoarthritis Outcome Score** **(HOOS; scores out of 100, lower = worse)**	
Pain	86.7 (16.7)
Symptoms	88.2 (13.7)
Quality of life	80.7 (22.0)
Activities of daily living	88.9 (14.5)
Sports	81.9 (24.1)
**Knee injury and Osteoarthritis Outcome Score** **(KOOS; scores out of 100, lower = worse)**	
Pain	88.3 (17.6)
Symptoms	67.4 (16.5)
Quality of life	77.1 (21.9)
Activities of daily living	90.2 (14.1)
Sports	77.1 (27.9)
**Participation in activities**	
Physical (hours/week)	2.94 (3.21)
Social (1 = infrequent, 5 = very frequent)	2.77 (0.56)
Cognitive (1 = infrequent, 5 = very frequent)	3.21 (0.58)
**Vascular diseases**	
Stroke	1 (2.0%)
Congestive heart failure	3 (6.1%)
Claudication	9 (18.4%)
Myocardial infarction	3 (6.1%)
Total number of vascular diseases reported (0 to 4)	0.33 (0.57)
**Vascular disease risk factors**	
Hypertension	27 (55.1%)
Diabetes mellitus	7 (14.3%)
Smoker (either current or former)	24 (49.0%)
Total number of vascular risk factors reported (0 to 3)	1.18 (0.83)

### Association of hip and knee ROMs with demographics

Hip and knee ROMs were correlated with each other (r = 0.67) and were both negatively correlated with age (hip/knee r = -0.55/-0.40). There were no associations of ROMs with education or sex. The correlations between hip and knee ROMs and demographics and sensor-derived mobility metrics are presented in Table 2.

**Table 2:** Correlations of joint motion metrics with demographics and mobility metrics.

Variable	Correlation with hip ROM (r, p)	Correlation with knee ROM (r, p)
**Knee ROM**	**0.67 (< .0001)**	-
**Demographics**		
Age at gait testing	**-0.55 (< .0001)**	**-0.40 (0.0045)**
Female	-0.05 (0.74)	0.23 (0.11)
Education	0.25 (0.081)	0.12 (0.39)
**Mobility measures (from wearable sensor on lower back)**		
Gait speed	**0.39 (0.013)**	**0.38 (0.016)**
Gait cadence	-0.14 (0.39)	-0.16 (0.32)
Gait step variability	0.15 (0.35)	-0.10 (0.56)
Gait stride regularity	**0.34 (0.017)**	-0.02 (0.88)
Transition 1 duration	**0.45 (0.0012)**	**0.51 (0.0002)**
Transition 1 range	**0.33 (0.021)**	**0.32 (0.026)**
Transition 1 median	**0.35 (0.014)**	**0.39 (0.0057)**
Transition 2 jerk	**0.31 (0.031)**	**0.29 (0.044)**
Transition 2 range	0.00 (1.0)	0.06 (0.71)
Turn yaw	**0.58 (< .0001)**	**0.38 (0.0074)**
Turn frequency	-0.011 (0.95)	-0.10 (0.53)
Standing with eyes closed sway	-0.19 (0.19)	**-0.30 (0.042)**

Correlation coefficients in boldface had p<0.05.

### Association of hip and knee ROMs with sensor-derived mobility metrics

Several mobility performance metrics derived from a sensor worn during the structured mobility testing session were associated with both hip and knee ROM, while others exhibited a more joint-specific association (Table 2). For example, there was stronger association of stride regularity with hip ROM than with knee ROM.

### Association of hip and knee ROMs with other mobility-related physical function and health measures

Total daily physical activity derived from a wrist-worn monitor was associated with hip ROM (r = 0.37) but not knee ROM (Table 3). A summary motor measure derived from 10 conventional motor tests was correlated with both hip and knee ROM (hip/knee r = 0.59/0.52). Parkinsonian gait had a negative correlation with hip and knee ROM (r = -0.57/-0.70), as did self-reported indicators of mobility disability (r = -0.51/-0.47) and fall history (r = -0.42/-0.32). BMI was not related to either ROM.

**Table 3:** Correlations of joint motion metrics with clinical and other measures.

Variable	Correlation with hip ROM (r, p)	Correlation with knee ROM (r, p)
**Actigraphy (wrist-worn monitor)**		
Total daily physical activity	**0.37 (0.029)**	0.26 (0.14)
**Other conventional motor and mobility-related clinical measures**		
Composite motor measure	**0.59 (< .0001)**	**0.52 (0.0001)**
Parkinsonian gait	**-0.57 (< .0001)**	**-0.70 (< .0001)**
Body mass index	-0.26 (0.074)	-0.20 (0.17)
Mobility disability	**-0.51 (0.0002)**	**-0.47 (0.0007)**
Fall history	**-0.42 (0.0029)**	**-0.32 (0.024)**
**Hip disability and Osteoarthritis Outcome Score**		
Pain	0.10 (0.51)	**0.31 (0.041)**
Symptoms	0.28 (0.063)	**0.37 (0.014)**
Quality of life	0.29 (0.053)	**0.45 (0.0019)**
Activities of daily living	0.28 (0.06)	**0.47 (0.0012)**
Sports	0.24 (0.17)	**0.34 (0.042)**
**Knee injury and Osteoarthritis Outcome Score**		
Pain	0.28 (0.061)	**0.44 (0.0025)**
Symptoms	0.17 (0.27)	0.22 (0.14)
Quality of life	0.27 (0.069)	**0.47 (0.0011)**
Activities of daily living	0.28 (0.061)	**0.40 (0.0067)**
Sports	0.12 (0.56)	0.28 (0.16)
**Participation in activities**		
Physical	0.047 (0.75)	0.084 (0.56)
Social	**0.29 (0.045)**	0.28 (0.055)
Cognitive	0.10 (0.47)	0.14 (0.35)
**Vascular diseases**		
Stroke	0.15 (0.29)	0.23 (0.11)
Congestive heart failure	-0.13 (0.38)	-0.07 (0.64)
Claudication	**-0.45 (0.0011)**	**-0.31 (0.030)**
Myocardial infarction	-0.22 (0.13)	-0.10 (0.48)
Total number of vascular diseases reported	**-0.44 (0.0016)**	-0.25 (0.089)
**Vascular disease risk factors**		
Hypertension	-0.23 (0.10)	-0.09 (0.55)
Diabetes mellitus	-0.14 (0.34)	-0.03 (0.82)
Smoker	0.09 (0.52)	-0.04 (0.78)
Total number of vascular risk factors reported	-0.14 (0.32)	-0.09 (0.53)

Correlation coefficients in boldface had p<0.05.

Knee ROM was correlated with all 5 domains of self-reported hip pain and dysfunction assessed by the HOOS (r = 0.31 to 0.47) and with 3 domains of self-reported knee pain and dysfunction from the KOOS (pain, quality of life, activities of daily living, r = 0.40 to 0.47). Hip ROM exhibited similar trends as knee ROM in its correlations with HOOS and KOOS domains, but these were not significant at the p<0.05 level (Table 3).

Of the different types of activity for which participants reported their level of engagement, it was the frequency of social activities rather than physical or cognitive activities that was most highly correlated with hip and knee ROMs (r = 0.29, Table 3).

Claudication but no other individual vascular diseases or risk factors were correlated with hip and knee ROM (hip/knee r = -0.45/-0.31), although the number of reported vascular diseases was negatively correlated with hip ROM (r = -0.44, Table 3).

## Discussion

We investigated the use of a portable, depth-sensing camera to quantify joint motion of older adults during mobility testing in the community. Using this camera, we successfully acquired three-dimensional motion data from nearly all 52 enrollees. Hip and knee ROMs extracted from the motion recordings were associated with mobility metrics acquired during the same testing session via a wearable sensor. In addition, joint motion metrics were related to a wide range of other mobility-related physical function and health measures. These results support the notion that a portable, depth-sensing camera can be used to expand instrumented gait testing conducted outside the laboratory setting. Further studies using this approach in larger numbers of older adults are needed to elucidate the independent contributions of joint motion and spatiotemporal mobility metrics to late life mobility impairments.

This work brings together two separate but related lines of research. Our group and others have successfully employed an unobtrusive wearable sensor for instrumented gait testing of older adults in the community setting [11,12]. Although a variety of mobility performance metrics can be extracted from recordings of this device, the single, belt-mounted sensor does not provide information about joint motion. Newer systems that incorporate multiple such devices worn on the legs and trunk are capable of tracking lower extremity joint motion, but these may not be well tolerated by older adults due to the extra time required for setup and calibration and the need to wear multiple tight-fitting elastic sleeves. In another line of research, investigators have explored the use of depth-sensing camera technology for gait analysis. However, with few exceptions [27,28], these studies have taken place in controlled laboratory environments [6–8,29–33]. Conversely, older adults participating in the current study were assessed while wearing everyday attire at their own residences by personnel who were trained in the use of the depth-sensing camera but otherwise inexperienced in motion capture technology and techniques. The high rate of successful data acquisition under these conditions suggests that the camera was not an overly burdensome addition to the single wearable sensor during the instrumented mobility testing session for either the study participants or the staff who administered the testing.

We also gleaned valuable information from the 3 cases in which we were not successful in obtaining hip and knee ROMs. In particular, two individuals withdrew from the study at the time of gait testing due to difficulties encountered by staff in setting up the depth-sensing camera system. Further refinements to reduce the required physical space may help decrease the refusal rate. One other participant’s atypical gait pattern (one foot placed directly in front of the other, obscuring it from view of the camera) precluded extraction of reliable hip and knee ROM values, indicating that the depth-sensing camera may fail to accurately track the biomechanics of a small subset of individuals due to certain aspects of their gait, body habitus, or other unforeseen factors.

We explored the potential clinical relevance of hip and knee ROM values obtained using the depth-sensing camera. Earlier studies concluded that these metrics were not accurate enough to serve as reliable gait metrics [7,8,30,31]. A recent review article affirmed these studies’ findings that angular measures of hip and knee motion were less accurate than spatiotemporal measures such as stride length. However, the review also suggested that in some instances, the benefits of a low-cost, portable, and user-friendly depth-sensing camera might outweigh the accompanying penalty in accuracy [9]. It is therefore noteworthy that in this work, in which data was collected in the community setting, hip and knee angular ROMs were correlated with several previously validated mobility metrics obtained using a belt-worn sensor during the same mobility testing session [11,12,34]. These metrics, which probe a diverse range of mobility dimensions but do not provide direct information on lower limb kinematics, exhibited statistically significant correlation coefficients ranging from 0.31 to 0.58 with hip ROM and from 0.29 to 0.51 with knee ROM (Table 2). One interpretation of these low- to moderate-strength correlations is that the hip and knee ROMs derived from the depth-sensing camera capture an additional facet of mobility that is related to, but not duplicative of, sensor-derived mobility metrics.

Furthermore, although this was a small pilot study with limited power, there were some indications of differential associations of hip and knee motion measures with the sensor-derived mobility metrics (Table 2). Specifically, stride regularity was correlated with hip ROM but not knee ROM, while sway during quiet standing with eyes closed was more strongly correlated with knee ROM. This preliminary work hints at the possibility of harnessing emerging technologies to uncover specific biologic substrates of mobility impairments. Further work in larger number of older adults is warranted to clarify the potentially independent associations of joint motion and sensor-derived mobility metrics with impaired mobility.

We found additional support for the clinical relevance of joint motion metrics in their associations with a wide range of other mobility-related phenotypes and measures. These included objective metrics of physical activity, several other metrics of motor function, self-report indicators of joint pain and dysfunction, mobility disability, and falls. These findings suggest that the portable depth-sensing camera system could help to uncover unique patterns of gait alteration that portend or cause specific phenotypes and mobility impairments that are common in later life.

This work diverges from the majority of earlier studies that utilized depth-sensing cameras for gait analysis in that we employed a third party developer’s software to carry out post hoc frame-by-frame body pose estimation rather than using the camera manufacturer’s software development kit (SDK) for real-time pose estimation [29,35,36]. This post hoc pose estimation software requires extra time and resources to process the data, but in the current work, this drawback was outweighed by an improvement in the consistency and accuracy of three-dimensional body tracking over the SDK’s real-time pose estimates. Thus, the use of post hoc pose estimation software might partially account for the current study’s findings of widespread association between hip and knee joint motion derived from depth-sensing camera recordings and mobility-related measures and phenotypes, many of which have not been previously reported. This technical detail may therefore have an important impact on the future clinical utility of depth-sensing camera technology. Work is underway to further automate the pose estimation processing as part of a high throughput system that can accommodate large data sets.

Overall, results of this study show that the depth-sensing camera is promising but has limitations. It cannot, for example, match the accuracy, temporal resolution, or functionality of a dedicated, laboratory-based gait analysis system. Such systems have very high temporal and spatial resolution and incorporate force plates to capture ground reaction forces, enabling determination of inverse kinematics. Bringing a force plate into the community setting or estimating ground reaction forces with wearable accelerometers are technically challenging endeavors that will require additional study in order to successfully integrate this data with the depth-sensing camera. Thus, we are currently limited to deriving kinematic measures from the depth-sensing camera in the community setting.

This work may have important implications for further aging research and clinical studies of mobility in both the short and long term. In particular, depth-sensing camera technology adds to a growing portfolio of cost-efficient, portable devices that are now available for more comprehensive and objective quantification of multiple dimensions of mobility and other diverse behaviors in large, community-based cohort studies of aging. By collecting three-dimensional kinematic data longitudinally and combining it with objective measures of mobility and habitual physical activity from other wearable sensors [11,12], investigators and clinicians can obtain a more comprehensive characterization of healthy mobility and impairments. This practice may help to identify certain alterations or combinations of alterations (beyond simple hip and knee ROMs) that specifically portend different types of mobility impairment and facilitate risk stratification of older adults, allowing early, targeted interventions. In addition to its utility in research, depth-sensing camera technology could eventually provide patients and physicians with wider access to quantitative mobility testing, either in a primary care clinic or via telemedicine in the comfort of the patient’s home. This conceptual paradigm for easier, lower cost, and more frequent gait testing using portable devices has the potential to impact clinical practice by facilitating the detection of early signs of mobility impairment, adding easily collected functional data on joint motion to treatment decision trees (e.g. regarding joint replacements), and monitoring the progression of gait alterations and their response to clinical interventions.

## Supporting information

S1 File Supplemental methods. (DOCX)
