# Supplementary material for: Expanding instrumented gait testing in the community setting: A portable, depth-sensing camera captures joint motion in older adults
Source: PLoS One. 2019 May 15;14(5):e0215995. doi: 10.1371/journal.pone.0215995 (PMC6519784; doi:10.1371/journal.pone.0215995)
Supplement: S1 File — (DOCX) [file pone.0215995.s001.docx]

**Supplemental methods**

**Depth-sensing camera: Expanded description of data collection and processing**

*Hardware and positioning:* During the structured mobility testing portion of each participant’s annual interview for the Rush Memory and Aging Project (MAP), the depth-sensing camera (Kinect for Windows, Microsoft Corporation, Redmond, WA) was positioned 4 feet beyond the 8-foot-long walking course for recording, as shown in Fig 2 of the main manuscript. For stability, the depth-sensing camera was placed on a relatively short tripod 20 inches above the ground. In order to capture the majority of the participant’s body as they approached the end of the walking course, the camera was angled upward slightly. Research assistants achieved a consistent angulation of 5 degrees by referencing a bubble level that was permanently attached to the camera’s housing. The camera was also rotated 90 degrees such that the wider dimension of its field of view was oriented in the vertical direction. In this positioning scheme, the 58.5 x 46.6-degree field of view intersected the ground plane and was approximately 53 inches tall at the end of the walking course and grew proportionally with increasing distance from the camera (see gray lines in Fig 2 of main manuscript). This positioning scheme was chosen over several other candidate schemes because it met the following criteria: a) the middle of the walking course was adequately covered by the field of view, such that one heelstrike-to-heelstrike gait cycle for each limb could be captured, b) its physical space requirements were compatible with the majority of testing environments in MAP, and c) it provided estimates of lower extremity motion that are similar in precision to estimates provided by any other positioning strategy.

*Data collection:* As the depth-sensing device’s infrared (IR) projector casts a pattern of IR light onto the field of view, an IR camera records this pattern from a vantage point that is shifted horizontally from the projector. The apparent horizontal displacement of each point in the IR light pattern compared to the true pattern depends on the distance between the sensor and the object captured in a given area of the field of view, and from this information a “depth image” is computed. Raw data were recorded from the device (color and depth video) at the maximum resolution (640 x 480), frame rate (30 frames per second), and quality settings possible using commercially available software (iPi Recorder version 3.1.1.34, iPi Soft, Moscow, Russia) running on a standard notebook computer (Pavilion 13 x360 Convertible PC, HP, Palo Alto, CA). The frame rate of the depth-sensing camera is variable and in some instances can fall below 30 frames per second, but using the equipment described above, the frame rate reported by the recording software never fell below 30 fps and we had no dropped frames in the current study.

*Data processing:* We transferred raw data from the notebook computer to a workstation (Z620, HP) for the computationally intensive frame-by-frame estimation of body pose using commercially available software (iPi Mocap Studio version 3.1.2.177, iPi Soft). We processed each dataset using the same routine. In particular, we first adjusted the habitus of a virtual human figure, including its height and approximate body mass index, to match those of the actual participant. We then manually selected a central frame of each walking performance and used the software’s Inverse Kinematics tool to approximately match the position of the virtual figure’s extremities to their real positions as visualized by the depth image. This provided an initial pose estimate. The automated pose estimation tool was then run backward and forward from the central frame, first in coarse mode and then in fine mode. The “Biomech” add-on for Mocap Studio was used to export the temporal course of body segment positions and joint angles in Matlab format. Then, custom graphic user interfaces (GUIs) and scripts written in Matlab were used to perform an additional round of quality control of every data set and to isolate one heelstrike-to-heelstrike gait cycle for each leg of a given participant for each walking trial. A heelstrike was defined as the maximum forward deviation of the foot relative to the pelvis. Within this gait cycle, we extracted the hip and knee range of motion (ROM) for each leg, defined as the difference between the maximum extension and the maximum flexion angles. According to variance component analysis of the data collected from 49 participants in the community setting, most of the variation in both the hip and knee ROMs was attributable to between-person variation rather than leg laterality or within-person (trial-to-trial) variation. We therefore averaged the four measurements (left and right legs from each of two traversals), yielding a mean hip ROM and a mean knee ROM for each participant.

*Comparison of depth-sensing camera with a state-of-the-art optoelectronic motion capture system:* We first enrolled 10 participants who were able to travel to and undergo testing in the Motion Analysis Laboratory at Rush University Medical Center. There, we employed an optoelectronic motion capture system (Qualysis, Gothenborg, Sweden) consisting of 12 infrared cameras, which together triangulated the position of 24 spherical markers affixed to the participants’ skin at locations prescribed by a modified Helen Hayes marker set, as previously described [1]. This yielded a 120-Hz reconstruction of the three-dimensional movement of the pelvis and lower extremities. Each participant performed 10 trials of walking along an 8-foot path at a self-selected speed while the lab-based system and the portable, depth-sensing camera acquired data simultaneously. After isolating one left and one right gait cycle per walking trial (heelstrike-to-heelstrike as described above) and aligning the depth-sensing camera data to the lab-based data via cross-correlation, we interpolated all gait cycles to 100 timepoints to facilitate comparisons. The two systems captured similar patterns of hip and knee flexion and extension throughout the gait cycle (Figs 1 and 2). For both the hip and knee, we computed the across-person standard deviation of the difference between the two systems at each point in the gait cycle (depth-sensing camera or lab-based system), and then averaged the corresponding variances across the cycle. We then converted to units of standard deviation to represent variation due to the device. We computed similar average variances for within-person (trial-to-trial) variation and for the variation due to differences in personal means. We noted that for both the hip and knee, the person-to-person variation was larger than either the variation due to device or the within-person, trial-to-trial variation (Table 1). Furthermore, the trial-to-trial variation was similar between the two systems. This comparison supports the idea that the depth-sensing camera captures hip and knee flexion and extension similar to that obtained with a state-of-the-art optoelectronic system.

**Table 1. Sources of variation in hip and knee angles.**

| **Source of variation** | **Standard deviation in hip angle (degrees)** | **Standard deviation in knee angle (degrees)** |
| --- | --- | --- |
| **System** (depth-sensing camera vs. lab-based) | 4.3 | 3.3 |
| **Trial-to-trial** (within-person) |  |  |
| Depth-sensing camera | 4.1 | 3.3 |
| Lab-based optoelectronic system | 3.2 | 3.9 |
| **Person-to-person** |  |  |
| Depth-sensing camera | 7.1 | 5.4 |
| Lab-based optoelectronic system | 4.5 | 4.6 |

| 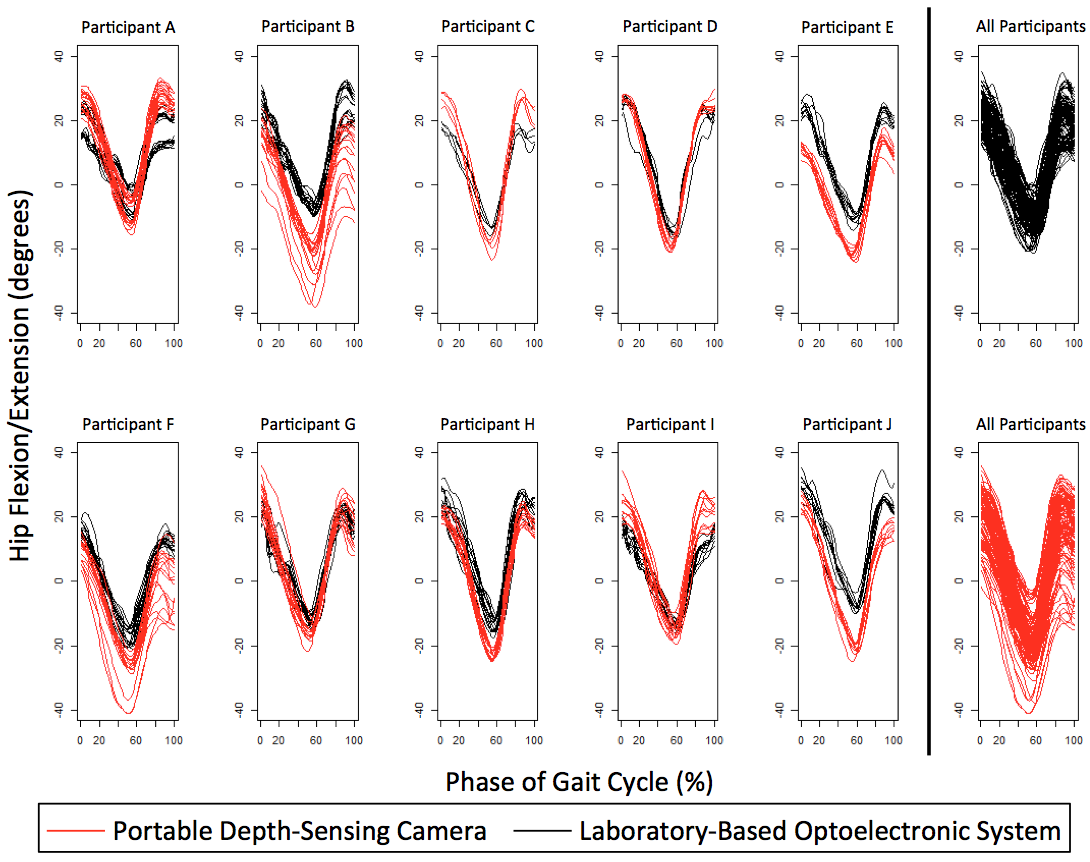 |
| --- |
| **Fig 1. Hip flexion and extension during gait cycles obtained using the depth-sensing camera (red) and state-of-the-art optoelectronic motion capture system (black).** Data are shown for individual participants from both systems (10 left panels) as well as for all participants from each system individually (2 right panels). |
| 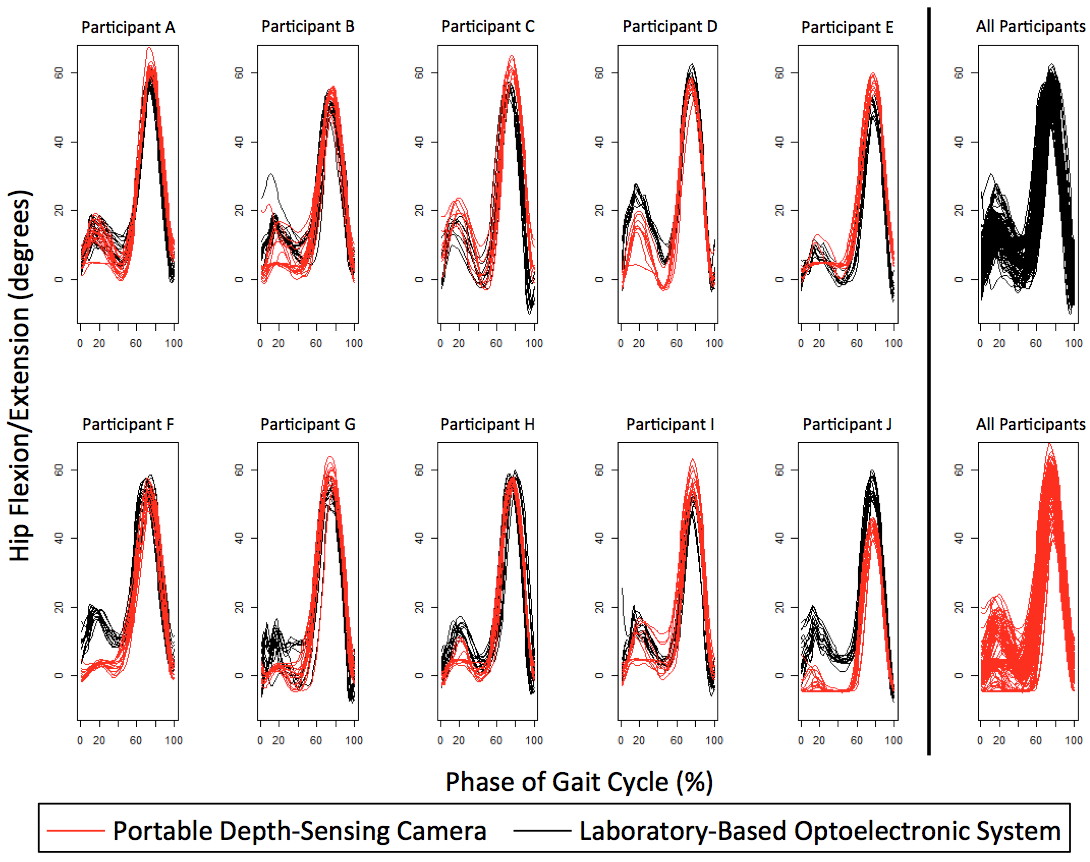 |
| **Fig 2. Knee flexion and extension during gait cycles obtained using the depth-sensing camera (red) and state-of-the-art optoelectronic motion capture system (black).** Data are shown for individual participants from both systems (10 left panels) as well as for all participants from each system individually (2 right panels). |

**Other mobility-related phenotypes and measures**

*Demographics*: Age at the time of mobility testing was computed based on the participant’s self-reported date of birth, which was recorded at MAP study entry along with sex and years of education.

*Total daily physical activity:* Participants wore a watch-sized activity monitor on the non-dominant wrist continuously for up to ten days. Movements of the wrist elicited a signal from the device’s embedded omnidirectional accelerometer, which was integrated with respect to time by the onboard processor, yielding an activity count for each 15-second epoch. Mean total daily physical activity was computed based on all available full days of recording, as previously described [2].

*Motor measures:* A summary motor measure was computed based on 10 conventional tests of gait, dexterity, and strength, as previously described [3]. A summary measure of the severity of parkinsonian gait signs was computed based on 6 items from a modified version of the United Parkinson’s Disease Rating Scale, as previously described [4].

*Body mass index (BMI):* BMI was calculated based on the participant’s height and weight measured at the time of mobility testing [5].

*Hip and knee joint pain and dysfunction:* Participants were asked to complete the Hip disability and Osteoarthritis Outcome Survey (HOOS) and the Knee injury and Osteoarthritis Outcome Survey (KOOS) while considering the hip or knee (left or right) that was giving them the most trouble at the time of mobility testing. These validated instruments assess self-reported hip and knee impairments on five subscales, each ranging from 0 (extreme symptoms) to 100 (no symptoms): pain, other symptoms, function in the activities of daily living (ADL), function in sports and recreation, and quality of life (QOL) [6,7].

*Mobility disability:* The Rosow-Breslau scale was used to assess mobility disability based on whether an individual reported being able to perform three activities independently: doing heavy housework, walking up and down stairs, and walking a half mile. Scores of 0 to 3 are possible and reflect the number of activities the participant requires help with or is unable to do, meaning that higher scores correspond to greater disability [8,9].

*Falls:* Fall history was based on participants’ self-report of having fallen at least once in the preceding year [10].

*Late-life activities:* Participants reported the frequency or duration of their engagement in specific physical, social, and cognitively stimulating activities, as previously described [11-13].

*Vascular risk factors and diseases:* Participants reported whether they had ever had a diagnosis of each of 4 vascular diseases (myocardial infarction, congestive heart failure, claudication, and stroke) as well as their exposure to 3 risk factors for vascular disease (hypertension, diabetes mellitus, and past or current smoking), as previously described [14,15].

**References**

1. Frank RM, Lundberg H, Wimmer MA, Forsythe B, Bach Jr BR, Verma NN, et al. Hamstring Activity in the Anterior Cruciate Ligament Injured Patient: Injury Implications and Comparison With Quadriceps Activity. Arthroscopy. 2016;32: 1651-1659.

2. Buchman AS, Boyle PA, Yu L, Shah RC, Wilson RS, Bennett DA. Total daily physical activity and the risk of AD and cognitive decline in older adults. Neurology. 2012;78: 1323-1329.

3. Buchman AS, Wilson RS, Leurgans SE, Bennett DA, Barnes LL. Change in motor function and adverse health outcomes in older African-Americans. Exp Gerontol. 2015;70: 71-77.

4. Buchman AS, Shulman JM, Nag S, Leurgans SE, Arnold SE, Morris MC, et al. Nigral pathology and parkinsonian signs in elders without Parkinson disease. Ann Neurol. 2012;71: 258-266.

5. Buchman AS, Wilson RS, Bienias JL, Shah RC, Evans DA, Bennett DA. Change in body mass index and risk of incident Alzheimer disease. Neurology. 2005;65: 892-897.

6. Roos EM, Lohmander LS. The Knee injury and Osteoarthritis Outcome Score (KOOS): from joint injury to osteoarthritis. Health Qual Life Outcomes. 2003;1: 64.

7. Nilsdotter AK, Lohmander LS, Klässbo M, Roos EM. Hip disability and osteoarthritis outcome score (HOOS)–validity and responsiveness in total hip replacement. BMC Musculoskelet Disord. 2003;4: 10.

8. Rosow I, Breslau N. A Guttman health scale for the aged. J Gerontol. 1966.

9. Buchman AS, Boyle PA, Wilson RS, Gu L, Bienias JL, Bennett DA. Pulmonary function, muscle strength and mortality in old age. Mech Ageing Dev. 2008;129: 625-631.

10. Buracchio T, Arvanitakis Z, Leurgans S, Bennett DA. Parkinsonian signs and incident falls in older persons without Parkinson’s disease. J Am Geriatr Soc. 2010;58: 205.

11. Buchman AS, Boyle PA, Wilson RS, Bienias JL, Bennett DA. Physical activity and motor decline in older persons. Muscle Nerve. 2007;35: 354-362.

12. Buchman AS, Boyle PA, Wilson RS, Fleischman DA, Leurgans S, Bennett DA. Association between late-life social activity and motor decline in older adults. Arch Intern Med. 2009;169: 1139-1146.

13. Wilson RS, Mendes De Leon CF, Barnes LL, Schneider JA, Bienias JL, Evans DA, et al. Participation in cognitively stimulating activities and risk of incident Alzheimer disease. JAMA. 2002;287: 742-748.

14. Boyle PA, Buchman AS, Wilson RS, Leurgans SE, Bennett DA. Association of muscle strength with the risk of Alzheimer disease and the rate of cognitive decline in community-dwelling older persons. Arch Neurol. 2009;66: 1339-1344.

15. Aggarwal NT, Bienias JL, Bennett DA, Wilson RS, Morris MC, Schneider JA, et al. The relation of cigarette smoking to incident Alzheimer’s disease in a biracial urban community population. Neuroepidemiology. 2006;26: 140-146.
